# Poly(amino carbonate urethane)-based biodegradable, temperature and pH-sensitive injectable hydrogels for sustained human growth hormone delivery

**DOI:** 10.1038/srep29978

**Published:** 2016-07-20

**Authors:** V. H. Giang Phan, Thavasyappan Thambi, Huu Thuy Trang Duong, Doo Sung Lee

**Affiliations:** 1 School of Chemical Engineering, Theranostic Macromolecules Research Center, Sungkyunkwan University, Suwon, Republic of Korea

## Abstract

In this study, a new pH-/temperature-sensitive, biocompatible, biodegradable, and injectable hydrogel based on poly(ethylene glycol)-poly(amino carbonate urethane) (PEG-PACU) copolymers has been developed for the sustained delivery of human growth hormone (hGH). In aqueous solutions, PEG-PACU-based copolymers existed as sols at low pH and temperature (pH 6.0, 23 °C), whereas they formed gels in the physiological condition (pH 7.4, 37 °C). The physicochemical characteristics, including gelation rate, mechanical strength and viscosity, of the PEG-PACU hydrogels could be finely tuned by varying the polymer weight, pH and temperature of the copolymer. An *in vivo* injectable study in the back of Sprague-Dawley (SD) rats indicated that the copolymer could form an *in situ* gel, which exhibited a homogenous porous structure. In addition, an *in vivo* biodegradation study of the PEG-PACU hydrogels showed controlled degradation of the gel matrix without inflammation at the injection site and the surrounding tissue. The hGH-loaded PEG-PACU copolymer solution readily formed a hydrogel in SD rats, which subsequently inhibited the initial hGH burst and led to the sustained release of hGH. Overall, the PEG-PACU-based copolymers prepared in this study are expected to be useful biomaterials for the sustained delivery of hGH.

Recent advances in protein therapeutics have garnered increasing attention for the treatment of various debilitating diseases^1^. Unlike small molecule drugs, proteins are naturally produced and well tolerated in the body after administration^2^. As a result, proteins that are highly specific with a complex set of functions can provide effective treatment, which might not be mimicked using small molecule drugs^2^. Despite these advantages, there are some limitations due to the intrinsic properties of proteins, including the short plasma half-life and poor absorption in gastrointestinal tracts, which led to the frequent injection that result in non-specific toxicity, renal failure, and poor patient compliance with increased cost. For instance, children with growth disorders require daily parenteral injection of human growth hormone (hGH) for a number of years^3^. hGH, composed of 191 amino acids, is synthesized and stored in the anterior pituitary gland, and it stimulates growth and production of insulin-like growth factor 1 (IGF-1)^4^. For stimulating the production of IGF-1 and insulin-like growth factor binding protein-3 (IGF-3), the minimum effective concentration of hGH was found to be 1–5 ng/mL^5^. Owing to its short plasma half-life (3.8–4 h)^6^, hGH is administered either daily or three times per week by subcutaneous injection, which leads to poor patient compliance^7^. Therefore, much attention has been paid to the development of a sustained hGH delivery system. Nutropin Depot, the first long-acting dosage form of hGH for a once-monthly or twice-monthly delivery formulation by a single injection, was prepared by the encapsulation of hGH into poly(lactic acid-*co*-glycolic acid) (PLGA) microspheres^8^. However, the formulation was later withdrawn from the market owing to its high cost in the scale-up process. In addition, denaturation of hGH during its production, which was possibly due to the use of organic solvents in the encapsulation procedure, inflammation around the injection site and the acidic environment generated by the local hydrolysis of PLGA further limit its application. To surmount these issues, delivery systems that could provide sustained protein delivery within the therapeutic window over an extended period should be developed.

Numerous studies have focused on the development of a hGH delivery system with the ultimate aim of decreasing the initial burst, and increasing the therapeutic duration and bioavailability^9^,^10^. In particular, PEGylation or crystallization of hGH has generally been used to control its delivery^6^,^7^,^11^,^12^,^13^. Alternatively, hGH can also be loaded into microspheres to control its delivery^14^. However, these systems have been limited by their initial burst release, low protein loading, and use of harsh preparation conditions, including organic solvent-based encapsulation methods often result in the loss of biological activity. In recent years, hydrogels, a three-dimensional porous network, composed of polymer blocks that can absorb or retain large quantities of water or biological fluids have been extensively studied^15^. Owing to the structural similarities between hydrogels and the extracellular matrix, proteins are easily encapsulated into the network without loss of biological activity^16^,^17^. Moreover, the soft nature of hydrogels reduces the inflammation at the site of administration. The traditional preformed hydrogels, however, lack *in situ* injectability and require surgical implantation that is rather costly and inconvenient to patients. Therefore, to overcome this shortcoming, attention has been given to injectable hydrogels in which aqueous solutions of precursor hydrogels can be injected using a small syringe which form a viscoelastic gel in the body^18^,^19^,^20^. The major advantages of injectable hydrogels are as follows: the high moldability because of their ability to form a desired shape at the defect site that is coherent with the surrounding tissues, the easy administration of gels to inaccessible sites because of their low viscosity, and *in vivo* delivery in a minimal invasive manner because of their small scar size formation during administration with less pain for patients^17^,^21^,^22^. These unique features are beneficial in reducing the recovery time and the risk of infection in patients. Therefore, injectable hydrogels have been extensively studied for a range of biomedical and pharmaceutical applications, including drug delivery and tissue engineering^21^,^23^,^24^,^25^.

Stimuli-sensitive injectable hydrogels, also termed smart hydrogels, which are responsive to various physical stimuli (e.g., light and temperature) and chemical stimuli (e.g., pH, ionic strength, and redox), provide several advantages when used in sustained release systems^26^,^27^,^28^,^29^,^30^,^31^. Thermo-sensitive hydrogels exist in a sol state at low temperature and facilitate interactions with therapeutic proteins, whereas they transform to a gel state at physiological temperature (37 °C). Various amphiphilic or hydrophilic polymers, such as poly(*N*-isopropyl acrylamide), pluronics, and poly(phosphazene), exhibit a sol-gel phase transition between room and body temperatures and are attractive materials for protein delivery^32^,^33^,^34^,^35^. However, the *in vivo* applications of these copolymers have been limited by their lack of biodegradability and their limited biocompatibility. Although the biocompatibility shortcoming has been surmounted using poly(ester)-based copolymers, which exhibit an ideal phase transition and biodegradability, the generation of acidic degradation products further limits their application *in vivo*. To surmount these limitations, poly(carbonate)-based copolymers have recently been studied for various biomedical applications^36^. Generally, poly(carbonate)-based copolymers are prepared through ring-opening of polymerization of trimethylene carbonate (TMC)^37^,^38^. The unique degradation behavior of poly(carbonate)-based copolymers, including their non-enzymatic hydrolysis, generation of weakly acidic biocompatible degradation products, and controlled surface erosion mechanism, open a new paradigm for applications in biomedical devices that could not be obtained with other polymers^36^,^37^,^39^. It is noteworthy that integration of poly(carbonate)-based copolymers with cationic or anionic polymers may provide useful hydrogel systems for the delivery of ionically charged proteins. For instance, poly(β-amino ester urethane), a cationic polymer, exists as a sol at certain temperatures, which facilitates the combination with anionic proteins through ionic interaction, but it can change to a gel at physiological temperatures and involved in sustained protein release^29^,^30^.

In this study, we developed biodegradable, cationic and injectable hydrogels composed of poly(ethylene glycol)-poly(amino carbonate urethane) (PEG-PACU) for the sustained delivery of hGH, a negatively charged model protein. The poly(β-amino carbonate) present in the copolymer is able to form an ionic complex with hGH, and the hydrophobic PACU enables the formation of a hydrogel network at body temperature (37 °C) (Fig. 1). The physicochemical characteristics of PEG-PACU were characterized using Fourier-transform infrared (FT-IR) spectroscopy and ^1^H NMR. The formation of an ionic complex between PEG-PACU and hGH was measured using the zeta potential, and sol-gel phase transitions were measured by the tube-inverting method. The *in vitro* cytotoxicity of PEG-PACU was tested through exposure to L929 cells. In addition, the *in vivo* gelation and the protein release behavior were evaluated in male Sprague-Dawley (SD) rats.

## Results and Discussion

**Figure 1:**
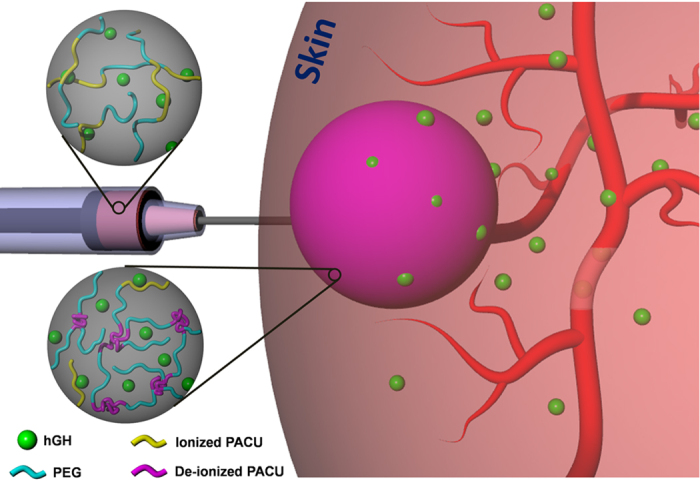
Schematic illustration of hGH-loaded PEG-PACU hydrogels and their sustained release characteristics after subcutaneous implantation.

In recent years, injectable hydrogels have been extensively studied for the sustained delivery of therapeutic proteins^22^,^32^. In this study, the unique sol-gel phase transition properties of PEG-PACU-based copolymers were utilized to design a biocompatible, biodegradable, and *in situ*-forming stimuli-sensitive injectable hydrogel system for the sustained delivery of hGH. The poly(amino) groups present in the PEG-PACU copolymer could form an ionic complex with the negatively charged hGH, as shown in Fig. 1. The copolymer/hGH complex was able to form a hydrogel depot after subcutaneous injection through a syringe, and the sustained degradation of copolymers disrupted the ionic and hydrophobic interactions, which allowed the sustained delivery of hGH.

### Synthesis and physicochemical characterization of PEG-PACU copolymers

**Figure 2:**
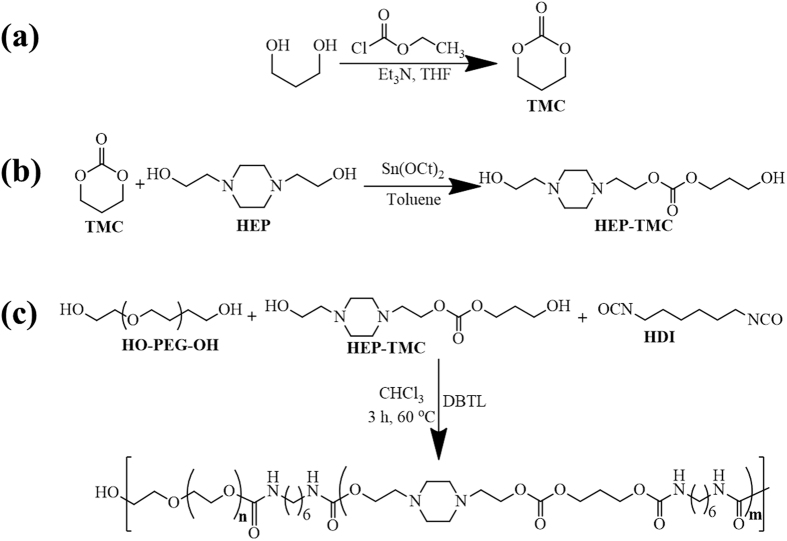
Synthesis of (**a**) TMC monomer, (**b**) HEP-TMC monomer, and (**c**) PEG-PACU copolymers.

The PEG-PACU copolymer was prepared via a three-step process, as shown in Fig. 2. For synthesis of pH-/temperature-sensitive PEG-PACU copolymers, TMC and amino carbonate (HEP-TMC) monomers were synthesized through cyclization and the ring-opening reaction, respectively. The reaction of 1,3-propanediol with ethyl chloroformate in the presence of base produced the cyclic carbonate TMC. The ^1^H NMR spectrum of TMC showed the characteristic methylene protons at 2.2 ppm (-CH_2_) and 4.33 ppm (-O-CH_2_) (Fig. S1). In the next step, HEP-TMC was synthesized through the ring-opening reaction of TMC initiated by the hydroxyl group of HEP. The peak at 2.55 ppm indicated the presence of a piperidine ring, and the peak at 4.33 ppm indicated the existence of a carbonate group as a part of the monomer. Finally, pH-/temperature-sensitive PEG-PACU copolymer was synthesized by the polyaddition polymerization reaction of HDI with HEP-TMC and PEG-diols. The chemical structures of the PEG-PACU copolymer were characterized using ^1^H NMR spectra (Fig. S1). The peak at 3.64 ppm belonged to the methylene units in the PEG, whereas the ^1^H signals at 3.15, 1.48 and 1.33 ppm were attributed to the methylene units of HDI in the copolymer. The peaks at 4.17, 2.61, 2.55, 4.24, and 1.98 ppm confirmed the presence of HEP-TMC in the final copolymer. The ^1^H NMR results demonstrated that both the monomer and the copolymer had been successfully synthesized.

The chemical structure of the PEG-PACU copolymer was further characterized using FT-IR spectra (Fig. S2a). The characteristic absorption bands at 3338 and 1533 cm^−1^ correspond to the stretching and bending vibrations of N-H in urethane groups, respectively. The peaks at 2941 and 2881 cm^−1^ are characteristic of the asymmetric and symmetric stretching vibrations of methylene groups. The strong bands at 1745 and 1685 cm^−1^ were assigned to the -C=O in the carbonate and urethane groups. The stretching frequency of the carbonate C-O was identified at 1249 cm^−1^. The C-O-C ether stretching vibration of PEG appeared at 1110 cm^−1^. In general, the ^1^H NMR and FT-IR spectra confirm the chemical structures of the PEG-PACU copolymer.

The M_n_ and PDI of PEG-PACU copolymers were measured by GPC. In Fig. S2b, the GPC traces of the reference PEG (M_n_ = 2050, Sigma-Aldrich) and the copolymer are presented, and the results are listed in Table 1. The GPC trace of the copolymer exhibited a relatively narrow molecular weight distribution and a shift toward a lower retention time, indicating a higher molecular weight than that of the reference PEG.

### Sol–gel phase transition diagram

**Figure 3:**
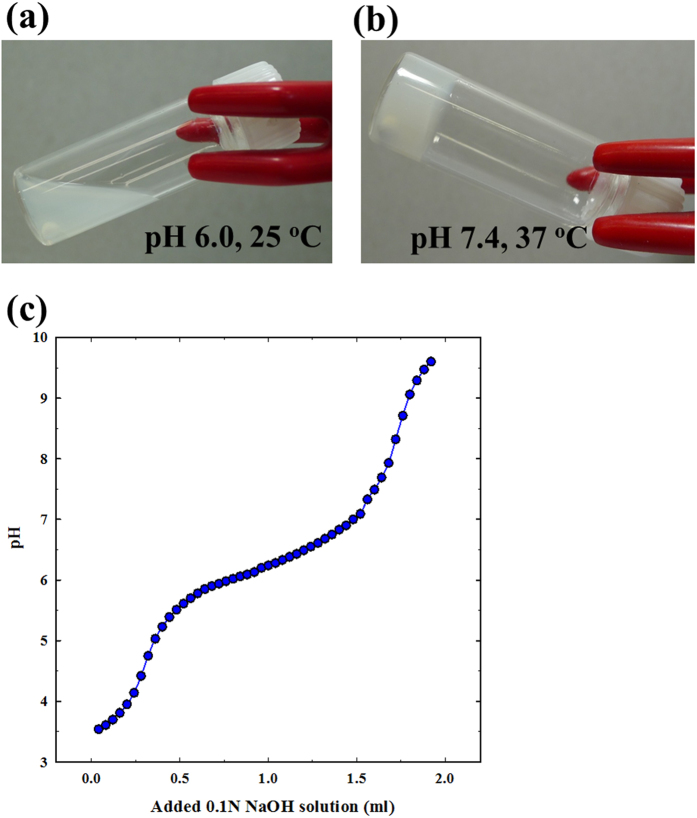
(**a**,**b**) Sol-gel phase transition photographs of PEG-PACU copolymers (25 wt%) as a function of pH and temperature and (**c**) Acid-base titration curve of PEG-PACU copolymers.

The sol-gel phase transition behavior of the PEG-PACU copolymer in aqueous solutions was monitored using the vial-inverting method^30^, and the copolymer exhibited a sol-gel phase transition as the pH and temperature increased. As shown in Fig. 3a,b, an aqueous copolymer solution of PEG-PACU exhibited a sol-to-gel transition in response to both pH and temperature changes, and the gel region covered the physiological condition. At low pH and temperature (e.g., pH 6 and 23 °C), the tertiary amine groups were ionized (pH < pK_a_), and the PEG-PACU copolymer become hydrophilic. Electrostatic repulsion between positively charged blocks also leads to a weak interaction between copolymer molecules and causes easy flow. These factors favor the sol state for the aqueous copolymer solution. In contrast, at a higher pH and temperature (e.g., pH 7.4 and 37 °C), mimicking the physiological condition, the tertiary amine groups almost became de-ionized, and the PACU blocks became hydrophobic. In addition, the hydrophobicity of the PACU blocks was further increased with increasing temperature because of the PEG dehydration. Consequently, the gel formed a network through hydrophobic interactions. In addition, the strength of the gel was also enhanced by hydrogen bonding due to the existence of urethane groups. For a more detailed explanation, the pH buffering property of the PEG-PACU was examined using acid-base titrations (Fig. 3c). From the acid-base titration tests, the pKa value of the copolymer, referred as the inflection point of the curve, was approximately 6.4. The titration curve of the PEG-PACU exhibited a considerable buffer capacity, thereby confirming the pH-sensitivity of the copolymer. The titration curve indicated that pKa was appropriate for physiological application.

### Rheological measurement

**Figure 4:**
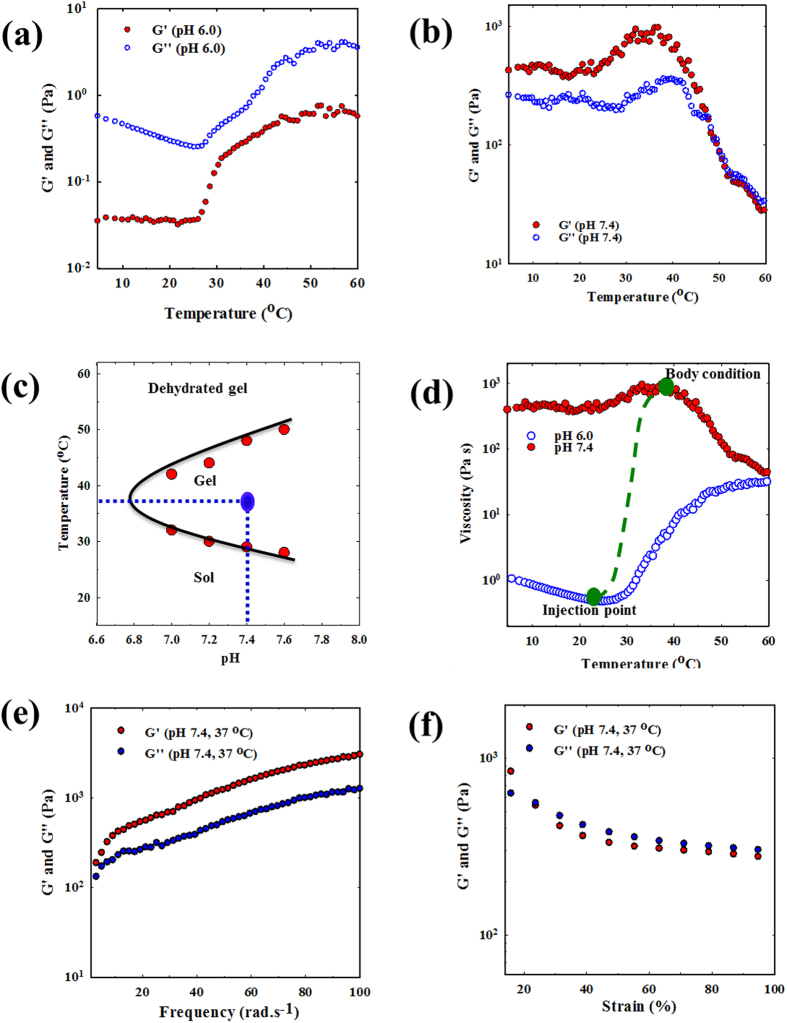
(**a**,**b**) Storage modulus (G′) and loss modulus (G″) of PEG-PACU copolymer hydrogels as a function of pH and temperature, (**c**) Sol-gel phase diagram of PEG-PACU copolymers as a function of pH and temperature, (**d**) Rheological properties of PEG-PACU copolymer hydrogels as a function of pH and temperature, and (**e**,**f**) The G′ and G″ were plotted against frequency and strain for PEG-PACU hydrogels; the tests were performed at a frequency of 6.28 rad.s^−1^. For all the tests the PEG-PACU copolymers concentration was kept at 25 wt%.

To determine whether the hydrogels could be useful in clinical application, the mechanical properties of the injectable hydrogels were examined. The rheological properties of a hydrogel system directly affect the mixing capacity of bioactive agents, the ability to be administered through a syringe, and the release behavior of the loaded therapeutics. Figure 4a,b presents the change in the storage modulus (G′) and loss modulus (G″) of the aqueous copolymer solution as a function of temperature. At pH 6.0, the magnitudes of both G′ and G″ were relatively small, and the value of G″ was greater than the G′ value in the surveyed temperature range. These results indicated a liquid state of the copolymer solution at pH 6.0. However, there was an abrupt increase in the G′ and G″ values at pH 7.4 compared with those at pH 6.0. At pH 7.4, the de-ionization of PACU segments led to the formation of a hydrogel network through hydrophobic interactions. The storage modulus was higher than the loss modulus in temperature range from 5–47 °C, exhibiting a typical elastic behavior^40^. Moreover, G′ was approximately 1.5 to 3 times larger G″ because the hydrogel properties mimicked those of *in vivo* soft tissue. When the temperature increased to approximately 40 °C, the values of both G′ and G″ rapidly decreased, suggesting a collapse in network structure of hydrogel (Fig. 4c). Because of the dehydration of the PEG segments and the greater packing in the hydrophobic domains, water gradually separated out of the hydrogel, leading to a condensed gel state^28^. At temperatures higher than 47 °C, G′ became lower than G″, and the hydrogel could not maintain its 3-dimensional network. The observed changes in G′ and G″ were consistent with a sol-gel transition investigation.

Furthermore, the change in viscosity with varying pH and temperature was monitored to evaluate the injectability of the system *in vivo*. Figure 4d shows the pH- and temperature-dependent changes in viscosity of the copolymer solution. At pH 6.0, the aqueous copolymer solution had relatively low viscosity (~0.5 Pa·s), which is appropriate for mixing with therapeutic agents and convenient for administration *via* a small syringe. However, at physiological conditions (pH 7.4 and 37 °C), the viscosity changed approximately 1800 times in magnitude, confirming the formation of a stable gel. Based on the viscosity profile, it is anticipated that the low-viscous PEG-PACU copolymer solution could be elegantly injected into an animal model through small gauge needles. The rapid phase transition at the physiological condition may allow the formation of a hydrogel depot.

In the frequency sweep experiment, G′ and G″ were plotted as a function of frequency. In the surveyed frequency range, G′ was always larger than G″, indicating a stable gel state at the physiological condition (Fig. 4e). Furthermore, the stability of hydrogel was determined from G′ and G″ versus strain. Figure 4f showed that both G′ and G″ decreased after a certain strain value. At value of strain approximately 24%, G′ became smaller G″, suggesting a collapse in network of hydrogel due to an excess deformation.

### *In vitro* cytotoxicity

**Figure 5:**
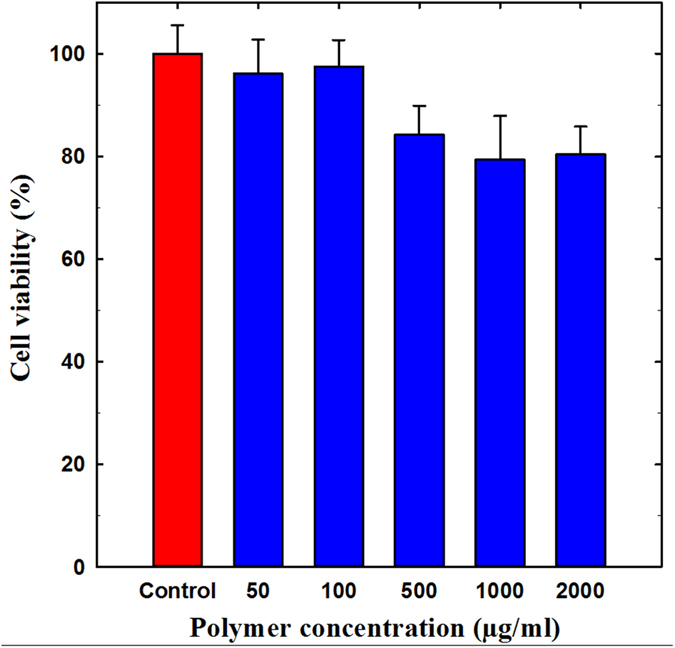
*In vitro* cytotoxicity of PEG-PACU copolymers. The error bars in the graph represent standard deviations (*n* = 5).

Prior to evaluating the gelation properties of PEG-PACU copolymer hydrogels in animal models, the biocompatibility of the copolymers was monitored using an MTT assay. To determine the *in vitro* cytotoxicity, the PEG-PACU copolymer was exposed to L929 cells at various concentrations (10 μg/mL to 2000 μg/mL) (Fig. 5). Irrespective of the polymer concentration tested, the cell viability was greater than 83%. In particular, most cells were viable up to a concentration of 2000 μg/mL. This cytotoxicity test clearly demonstrates that the PEG-PACU-based hydrogels prepared in this study can be used as non-cytotoxic implant materials for *in vivo* applications. Our MTT assay results implied that PEG-PACU-based hydrogels have low cytotoxicity, good biocompatibility and promising potential for biomedical applications, such as protein carrier.

### *In vitro* release and stability

Figure S3 shows the *in vitro* release of hGH from PEG-PACU hydrogels in PBS (pH 7.4, 37 °C) as a function of time. In physiological conditions (pH 7.4), hGH-loaded PEG-PACU hydrogels released 28% of the hGH in the first day. After which, release rate was slowly decreased, implying the control release of PEG-PACU hydrogels. It is noteworthy that the release rate was markedly slower after four days. Particularly, after five days PEG-PACU hydrogels released 81% of hGH. Unlike preformed hydrogels, the *in vitro* sustained release was not significant, perhaps because the property that injectable hydrogels are less stable under *in vitro* conditions; however, they are stable under *in vivo* conditions. The strong *in vivo* gelation of PEG-PACU copolymers is mainly due to the interaction of hydrophilic copolymers with biological fluids^41^. The extracellular proteins, including, cytokines, lipoproteins, and enzymes, can interact with hydrophilic copolymers at the extracellular matrix^41^,^42^.

In addition, to demonstrate the biological activity of hGH, we examined the reverse-phase high performance liquid chromatography (HPLC) analysis of released hGH (Fig. S4). The HPLC trace indicated that the native hGH was detected at 14 min. Interestingly, the hGH released from the injectable hydrogel was also appeared at an identical retention time. These results indicated that hGH released from PEG-PACU-based injectable hydrogels was in its native state.

### *In vivo* gelation and biodegradation

**Figure 6:**
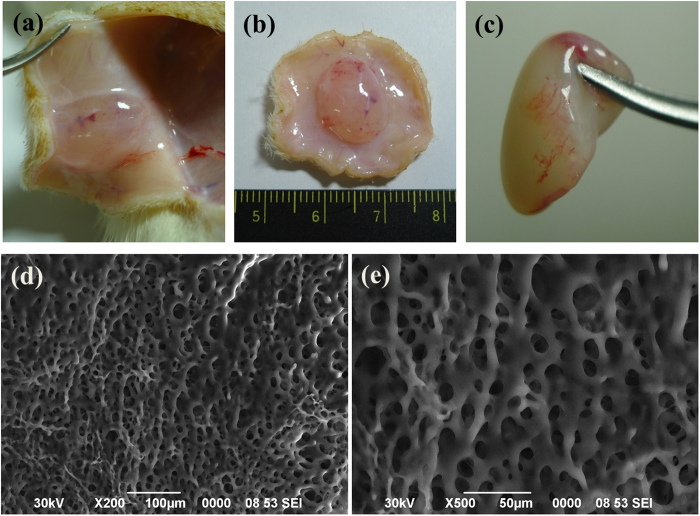
(**a–c**) *In vivo* gelation of PEG-PACU copolymers in the male SD rats. SD rates were sacrificed 10 min after subcutaneous administration of the PEG-PACU copolymer solution (25 wt%) and (**d**,**e**) Morphology of *in vivo* gels after lyophilization.

To examine the *in vivo* injectability and gelation of PEG-PACU copolymers, they were subcutaneously injected into the back of SD rats. 10 min after the subcutaneous injection of the copolymer solution, the rats were sacrificed to evaluate the mechanical properties and morphology of the gel *in vivo*. As shown in Fig. 6a–c, the PEG-PACU copolymers could form a gel with a well-defined shape and structure without inducing any inflammation. By varying the PEG-PACU copolymer concentrations from 10 wt% to 25 wt%, the precursor solution at this concentrations are easily flowed through the 26G needle and formed a hydrogel depot in the back of SD rats. The morphology of the *in vivo* gel was examined using SEM. Figure 6d,e demonstrated that the PEG-PACU copolymer could form a uniform porous structure, which indicated that the hydrogel properties were not altered after administration into the animal models.

**Figure 7:**
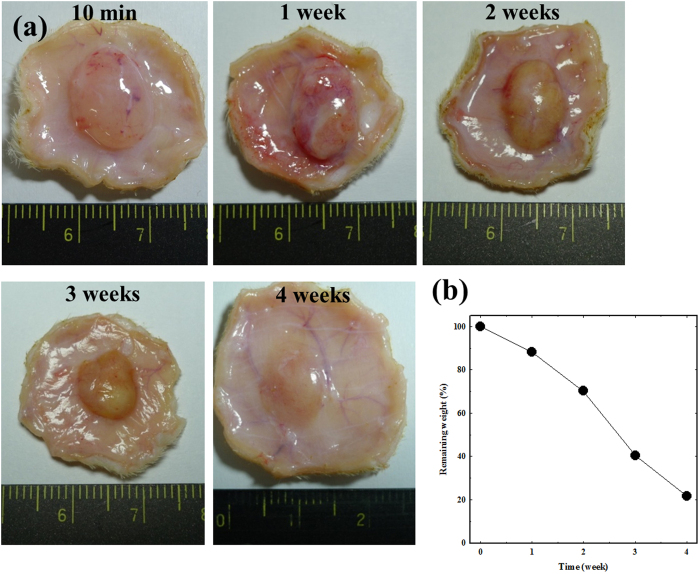
(**a**) Optical micrograph images *in vivo* biodegradation of PEG-PACU hydrogels (25 wt%) and (**b**) degradation rate was determined by the mass loss method.

The *in vivo* biodegradability of the PEG-PACU hydrogels was investigated by subcutaneous injection of the solution; subsequently, the rats were sacrificed 7, 14, 21, and 28 days after implantation. As shown in Fig. 7, by visual examination, we observed that degradation began at 7 days, with 10% of hydrogels were degraded. The gel lost approximately 30% of its weight in two weeks via erosion degradation. After two weeks, the M_n_ of the copolymer decreased, leading to a decrease in the gel rigidity, and a faster degradation rate was observed. After 4 weeks, the remaining gel was approximately 20%. The hydrogels were completely degraded after 5 weeks. More importantly, no inflammation was observed throughout the experiment. The results demonstrated that the copolymer is biodegradable and can be biologically metabolized. During the experiment period, there were no signs that the hydrogels were toxic to the skin.

### Zeta potential measurement

**Figure 8:**
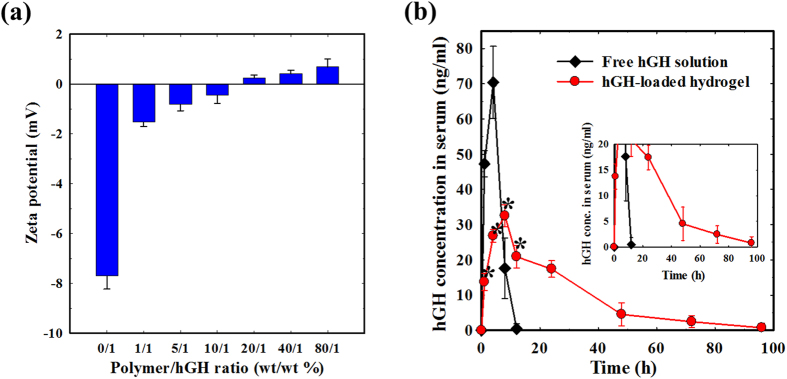
(**a**) Zeta potential of PEG-PACU copolymers with different hGH concentrations at pH 7.4 and (**b**) hGH concentration in the serum of SD rats at varying time points after the subcutaneous administration of 300 μL of free hGH solution and hGH-loaded PEG-PACU hydrogel formulation (*n* = 3). Asterisk (*) denotes statistically significant differences (p < 0.05) compared with free hGH solution.

The major objective of our study is to develop PEG-PACU-based cationic hydrogels for the sustained delivery of certain charged proteins. In this study, we chose hGH as an anionic model protein because its applications have been limited by its loss of bioactivity during systemic administration, thereby necessitating frequent injections. Therefore, the development of a suitable hydrogel formulation for the sustained delivery of hGH is necessary. The existence of electrostatic interactions or complexation between a PEG-PACU copolymer and hGH was examined by zeta potential measurement. Figure 8a shows the zeta potential value of the complex between the PEG-PACU copolymer and hGH at varying concentrations. As the PEG-PACU copolymer concentration in the polymer-protein complex increased, the zeta potential of the conjugates increased. The zeta potential of the pristine hGH solution was −7.8 mV, indicating the anionic nature of the protein, but it increased rapidly when hGH was mixed with the copolymer. The zeta potential was −1.53 mV at the 1/1 ratio and reached +0.68 at the 80/1 ratio. The finding indicates the formation of a good complex between the copolymer and hGH. This result indicated that the loaded hGH is effectively complexed by the poly(amine) present in the PEG-PACU copolymer, which may enable a decrease in the burst release of hGH.

### *In vivo* release of hGH

To examine the *in vivo* release pattern of hGH from the PEG-PACU hydrogels, we performed a pharmacokinetic study in SD rats. Based on the previous studies, it was found that the effective concentration of hGH in humans is appeared to be 1 to 5 ng/ml^35^,^43^,^44^. hGH is a single chain peptide hormone composed of 191 amino acids, and is secreted by the anterior pituitary gland that stimulates growths and cell production^5^. Prior to the *in vivo* test, the hGH was immobilized using PEG-PACU hydrogels, and its surface properties were tuned for sustained release, as observed in Fig. 8a.

The *in vivo* release of hGH was evaluated in two groups of SD rats (Fig. 8b). Namely, the hGH-loaded PEG-PACU hydrogel formulation and free hGH solution was used as control. As expected, the free hGH solution rapidly disappeared within 12 h of subcutaneous injection. In particular, a burst release was observed until 2 h, and then the hGH concentration in the plasma rapidly decreased. Interestingly, the hGH-loaded PEG-PACU hydrogel had a lower initial burst and significantly extended the release period of hGH compared with those of the free hGH solution. The existence of ionic interactions and hydrogen bonding between the hGH and PACU hydrogels in the formulation, due to the presence of poly(amine) and urethane groups, resulting in a reduced initial burst. Owing to the unique biodegradation characteristic of PEG-PACU hydrogels, as observed in Fig. 7, the hGH encapsulated within the hydrogel matrix showed an enhanced half-life, and the protein was released in a sustained manner over 4 days. Pharmacokinetic parameters, including maximum plasma concentration (C_max_), time point of maximum plasma concentration (T_max_), and area under the plasma concentration time curve (AUC), were suggested that the hGH-loaded hydrogel formulation was absorbed slower with considerable reduction of initial burst (Table S1 and Fig. 8b). The AUC values also indicated a higher bioavailability of the hGH-loaded hydrogel formulation than free hGH solution. These results indicated that hGH-loaded PEG-PACU hydrogels have the potential to be used as a sustained delivery system for hGH with a controlled initial burst.

## Conclusion

A well-defined PEG-PACU-based pH-/temperature-sensitive injectable hydrogel was synthesized, which could load hGH effectively and deliver in a sustained manner. An aqueous copolymer solution of PEG-PACU exhibited a sol-to-gel transition in response to both pH and temperature. The gel window well covers the physiological region in which the flowing sols formed a gel at 37 °C. The mechanical properties of PEG-PACU hydrogels are tunable through variations in the polymer concentration, pH and temperature. The cytotoxicity study indicated that the hydrogel was safe for *in vivo* applications. Low-viscous PEG-PACU sols at room temperature allowed the subcutaneous injection of a copolymer solution into the back of SD rats, and the solution formed a stable gel and biodegraded in 5 weeks without inflammation at the injection site. As a result, hGH-loaded PEG-PACU hydrogels-induced retardation of the initial burst and the hGH release was markedly increased compared with those of hGH alone. In particular, a single subcutaneous injection of hGH-loaded PEG-PACU hydrogels resulted in an effective hGH therapeutic level until 4 days. These results suggest that the PEG-PACU-based biodegradable and pH-/temperature-sensitive hydrogel prepared in this study can be used as a promising carrier for the sustained delivery of hGH with improvements in the stability, patient compliance, and inhibition of the burst release.

## Methods

### Materials

α,ω-Bis-hydroxy-poly(ethylene glycol) (HO-PEG-OH, M_n_ = 2050 g/mol), hexamethylene diisocyanate (HDI), stannous octoate (Sn(Oct)_2_), dibutyltin dilaurate (DBTL), 1,4-bis(2-hydroxyethyl)piperazine (HEP), anhydrous chloroform, anhydrous toluene, and phosphate-buffered saline (PBS) were purchased from Sigma-Aldrich Co. (St. Louis, MO, USA). Diethyl ether, n-hexane, sodium hydroxide (NaOH, 5 N), and hydrochloric acid (HCl, 5 N) were obtained from Samchun Co. (Seoul, Korea). All other chemicals were of analytical grade and used as received. The water (18.2 MΩ cm resistivity at 25 °C) used in the experiments was prepared using a Milli-Q water purification system.

### Synthesis of PEG-PACU copolymers

The PEG-PACU copolymer was prepared via a three-step process, as shown in Fig. 2.

### Synthesis of trimethylene carbonate (TMC)

The six-membered cyclic carbonate was synthesized from 1,3-propanediol using ethyl chloroformate as a ring-closing reagent. In brief, 1,3-propanediol (0.1 mol) was stirred at 0 °C in 200 ml of THF with a stoichiometric amount of trimethylamine (0.21 mmol) as a catalyst. Ethyl chloroformate (0.2 mol) was added to the solution, and the solution was then stirred for 12 h at room temperature. The reaction mixture was then filtered to remove the precipitated triethylammonium chloride, and the filtrate was concentrated under reduced pressure. The residue was recrystallized using THF/hexane (1 v/10 v), and the cyclized product was obtained as white crystals.

### Synthesis of HEP-TMC monomer

The HEP-TMC monomer was synthesized by the ring-opening reaction of TMC in the presence of HEP as an initiator and Sn(Oct)_2_ as a catalyst (Fig. 2). Briefly, TMC (20 mmol), HEP (20 mmol) and Sn(Oct)_2_ (0.4 mmol) were placed in a two-neck round-bottom flask and dried under vacuum at 50 °C for 4 h. Subsequently, 55 ml of anhydrous toluene was added to dissolve the reactants, the temperature was slowly increased to 120 °C, and the mixture was stirred for 24 h under dry nitrogen. The reaction mixture was then cooled to room temperature, precipitated in an excess of n-hexane, filtered, and dried under vacuum for 48 h to obtain HEP-TMC monomer.

### Synthesis of PEG-PACU copolymers

The PEG-PACU copolymer was synthesized by the polyaddition polymerization reaction^45^. Briefly, PEG (1 mmol) and DBTL (0.04 mmol) were added to a two-neck round-bottom flask and dried under vacuum at 110 °C for 2 h. Subsequently, the temperature was decreased to 60 °C, and monomer TMC-HEP (15 mmol) was added and dried under vacuum for 1 h. The reactant was further cooled under dry nitrogen, and 85 ml of anhydrous chloroform was added to dissolve the reactants. Finally, HDI (16 mmol) was added, and the reaction was conducted at 60 °C for 3 h. The flask was then cooled to room temperature, and the sample was precipitated using an excess of diethyl ether. The precipitated product was filtered and dried at room temperature under vacuum for 48 h.

### Characterization

The chemical structures of the monomer and polymers were characterized by ^1^H NMR (Varian Unity Inova 500NB) operating at 500 MHz. The chemical shifts are reported in ppm with the solvent proton signal as the internal reference. FT-IR spectra were obtained using an FTIR-ATR spectrometer (IFS-66/S, Bruker, USA). The spectra were recorded in the range between 600–4000 cm^−1^ with a resolution of 4 cm^−1^.

The average molecular weight (M_n_) and the polydispersity index (PDI) of the copolymer were measured by gel permeation chromatography (GPC) using a Waters Model 410 equipped with a refractive index detector (Shodex, RI-101) and three Styragel (KF-803, KF-802.5, and KF-802) columns in series. The flow rate of the CHCl_3_ eluent was 1.0 mL/min at 35 °C. Poly(ethylene glycol)s standards, with molecular weights ranging from 1400–20,600 g/mol, were used to calibrate the column.

The zeta potential of the copolymer, protein and copolymer-protein complex was measured using a Zeta-potential & Particle Size Analyzer (Malvern Instruments Inc., Massachusetts, USA). The zeta potential values were derived from electrophoretic mobility values using the Smouluchowski equation^45^,^46^. When the difference between the measured and calculated baselines was less than 0.1%, the correlation function was accepted. Before the zeta potential measurement, the solutions were stabilized at room temperature for 20 min. For zeta potential measurement, hGH was mixed with copolymer solutions at pH 6.0; after 1 h of stirring, the pH of the complexes were adjusted to 7.4 by 1 N NaOH. The hGH concentration was maintained at a constant value of 1.0 mg/ml.

A scanning electron microscope (SEM, JEOL JSM-6390) was utilized to visualize the cross-sectional morphology of the *in vivo* gels after lyophilization.

The pH-sensitivity of the PEG-PACU copolymer was measured using an acid-base titration method. In brief, 40 ml copolymer solution of PEG-PACU (2 mg/ml) in deionized water was adjusted to pH 4.0. The change in pH was monitored by the addition of 0.1N NaOH solution (40 μL).

### Sol–gel phase transition diagram

The sol (flow) and gel (non-flow) states of the copolymer in aqueous solutions were determined using the vial-inverting method^30^. In brief, the copolymer was dispersed in PBS solution at a 25 wt% concentration, and the pH was adjusted to 1.0 to obtain a clear solution. Subsequently, the pH was adjusted to specified values using 5 N NaOH or 5 N HCl. The samples with different pH values (~0.5 ml in a 5 mL vial) were placed in a temperature-controlled water bath, which was slowly heated from 2 to 60 °C with an equilibration time of 20 min for 2 °C intervals. The sol–gel behavior was determined by inverting the vials and observing the flow and non-flow states after 1 min.

### Dynamic rheological measurement

The rheological properties of the copolymer solutions at different pH values were evaluated by measuring the change in viscosity, storage modulus, and loss modulus with temperature using a dynamic mechanical analyzer (Bohlin Rotational Rheometer) in oscillation mode. The samples were placed between a 20 mm diameter upper plate and a 100 mm diameter bottom plate with a gap size of 0.25 mm, and the data were collected using a controlled shear stress of 0.4 Pa and a frequency of 1 rad s^−1^. The complex viscosity, storage modulus, and loss modulus were recorded as a function of temperature from 5 °C to 60 °C with a heating rate of 2 °C per min.

In addition, the stability of hydrogel at the physiological condition (37 °C, pH 7.4) was investigated through measuring the change of G′ and G″ as functions of frequency and strain. The frequency sweep test was performed in frequency range of 1–100 rad.s^−1^ under controlled strain of 0.1%, and the strain sweep test was surveyed with strain from 0.1% to 100% at a constant frequency of 6.28 rad.s^−1^.

### *In vitro* cytotoxicity

L929 cells, obtained from the American Type Culture Collection (Rockville, MD, USA), were used to examine the compatibility of the hydrogel formulation. Cells were cultured in DMEM (Gibco, Grand Island, NY, USA) containing 10% (v/v) heat-inactivated fetal bovine serum (FBS) and 1% (w/v) penicillin-streptomycin at 37 °C under 5% CO_2_-95% air atmosphere. The cells were seeded at a density of 1 × 10^4 ^cells/well in a 96-well flat-bottomed plate. After a day of growth, the cells were washed twice with PBS (pH 7.4) and incubated for 24 h with various concentrations of PEG-PACU copolymer. The cells were then washed twice with PBS to remove any remaining polymer, and fresh culture medium was added. 20 μL of 3-(4,5-dimethylthiazol-2-yl)-2,5-diphenyltetrazolium bromide (MTT) solution (5 mg/ml in PBS) was added to each well, and the cells were incubated for an additional 3 h at 37 °C. The culture medium was then removed and washed twice with PBS. Subsequently, the cells were dissolved in DMSO and the absorbance was measured using a BioTek microplate reader (Seoul, Korea) at 490 nm wavelength.

### *In vitro* release of hGH

For the *in vitro* hGH release experiment, the PEG-PACU copolymer solution (25 wt%) was mixed with hGH, at a final hGH concentration of 1 mg/ml, at pH 6.5. Then, 1 g of the sample solution was transferred to a vial, and the pH of the solution was adjusted to 7.4 and kept at 37 °C to obtain a gel. Three ml of PBS containing 0.02 wt% sodium azide was added on to the hydrogel containing vial. At predetermined time intervals, 1.5 ml of the medium was withdrawn and replaced with an equal amount of fresh medium. The hGH concentration was estimated using a bicinchoninic acid (BCA) assay kit (Thermo Fisher Scientific, IL, USA).

Furthermore, to investigate the stability of hGH, the protein released from hydrogels was analyzed using a HPLC. Waters system model 1525 equipped with a Waters 2489 UV/Visible detector and TSK2000SWXL column was used. The eluent was PBS pH 7.0 with 10% isopropanol at 30 °C and the detection was performed with maximum absorption at 215 nm.

### *In vivo* experiment

The *in vivo* gelation and biodegradation of PEG-PACU copolymers were evaluated in adult male Sprague-Dawley (SD) rats (220–250 g) supplied by the Korea Research Institute of Bioscience and Biotechnology (Daejeon, Korea). All experiments with live animals were performed in compliance with relevant laws and the institutional guidelines of the Sungkyunkwan University. The Sungkyunkwan University institutional committees approved the experiments. To examine the *in vivo* gel formation and the *in vivo* biodegradation of the copolymer, samples were subcutaneously injected through a 26-gauge needle into the back of SD rats. In brief, the aqueous copolymer solution was prepared at a concentration of 25 wt%, and 300 μL of the sample solutions was subcutaneously injected in the backs of SD rats. At designed time, the SD rats were sacrificed to examine the state of the *in vivo* gels and the surrounding tissue. The gels were isolated and freeze-dried for 3 days to determine the degradation of the gels.

### *In vivo* release of hGH

The hGH was chosen as a model protein to demonstrate the potential of the hydrogel as a great vehicle for sustained release of protein. For the *in vivo* release of hGH, the SD rats were randomly divided into 2 groups (3 rats/group). In each group, 500 μL hGH-loaded copolymer solution (hGH 3 mg/mL, 25 wt% copolymer) or 500 μL hGH solution (3 mg/mL) was subcutaneously injected into the back of SD rats. At predetermined time intervals, blood samples were withdrawn from the tail vein and centrifuged to obtain the sera, which were stored at −21 °C until the assay. The hGH concentration in the sera was analyzed using an immunoenzymetic assay kit (hGH-EASIA, DIAsource ImmunoAssays, Belgium).

### Statistical analysis

The values are expressed as the mean ± standard deviation. Statistical analysis was performed using the one-way ANOVA test. Differences were considered statistically significant if p < 0.05 (*).

## Additional Information

**How to cite this article**: Phan, V. H. G. *et al*. Poly(amino carbonate urethane)-based biodegradable, temperature and pH-sensitive injectable hydrogels for sustained human growth hormone delivery. *Sci. Rep.* **6**, 29978; doi: 10.1038/srep29978 (2016).

## Supplementary Material

Supplementary Information

## Figures and Tables

**Table 1:** Physiochemical characteristics of PEG-PACU copolymers.

Name	M_n_^a^	Copolymer structure[EGn-ACUm]_x_^b^	PACU (wt%)^c^	M_n_^d^	PDI^e^
PEG-PACU	2050	[EG_46_-ACU_11.5_]_0.95_	71	6780	1.71

^a^Collected from Sigma-Aldrich, ^b–d^Calculated using ^1^H NMR spectra, and ^e^Measured from GPC.
