# Supplementary material for: Poly(amino carbonate urethane)-based biodegradable, temperature and pH-sensitive injectable hydrogels for sustained human growth hormone delivery
Source: Sci Rep. 2016 Jul 20;6:29978. doi: 10.1038/srep29978 (PMC4951706; doi:10.1038/srep29978)
Supplement: Supplementary Information [file srep29978-s1.pdf]

(Supplementary Information for *Scientific Reports*)

Poly(amino carbonate urethane)-based biodegradable, temperature and pH-sensitive injectable hydrogels for sustained human growth hormone delivery

V.H. Giang Phan,[1] Thavasyappan Thambi,[1] Huu Thuy Trang Duong and Doo Sung Lee*

School of Chemical Engineering, Theranostic Macromolecules Research Center,

Sungkyunkwan University, Suwon, Republic of Korea

[1]These authors contributed equally contributed to this paper.

*Corresponding author:

Doo Sung Lee, Ph.D.

Tel.: +82-31-290-7282; Fax: 82-31-292-8790; e-mail: dslee@skku.edu

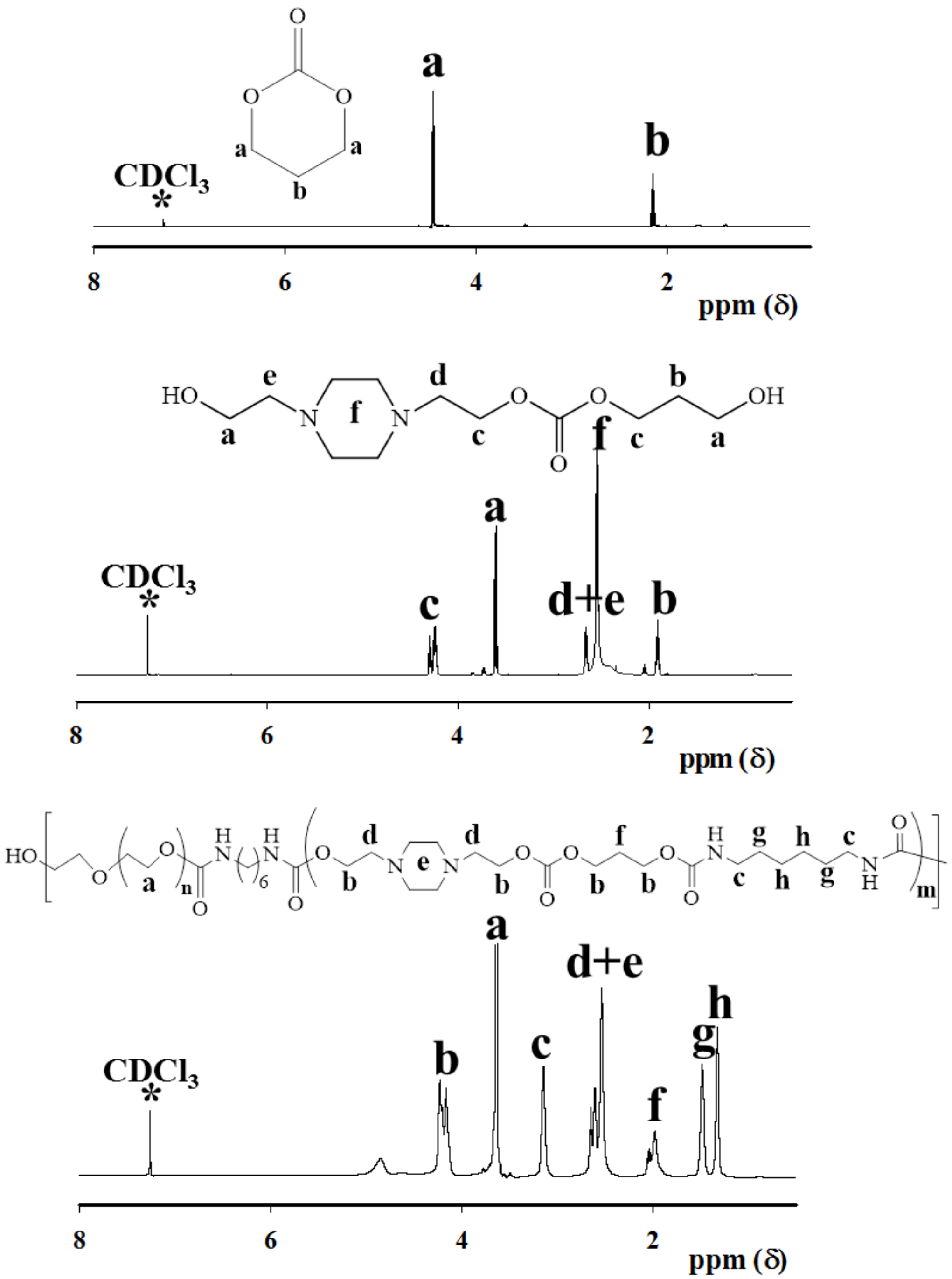

Fig. S1. ^{1}H NMR spectra of TMC and HEP-TMC monomers and PEG-PACU copolymers. Asterisks (*) indicates the residual solvent peak $CDCl_3$.

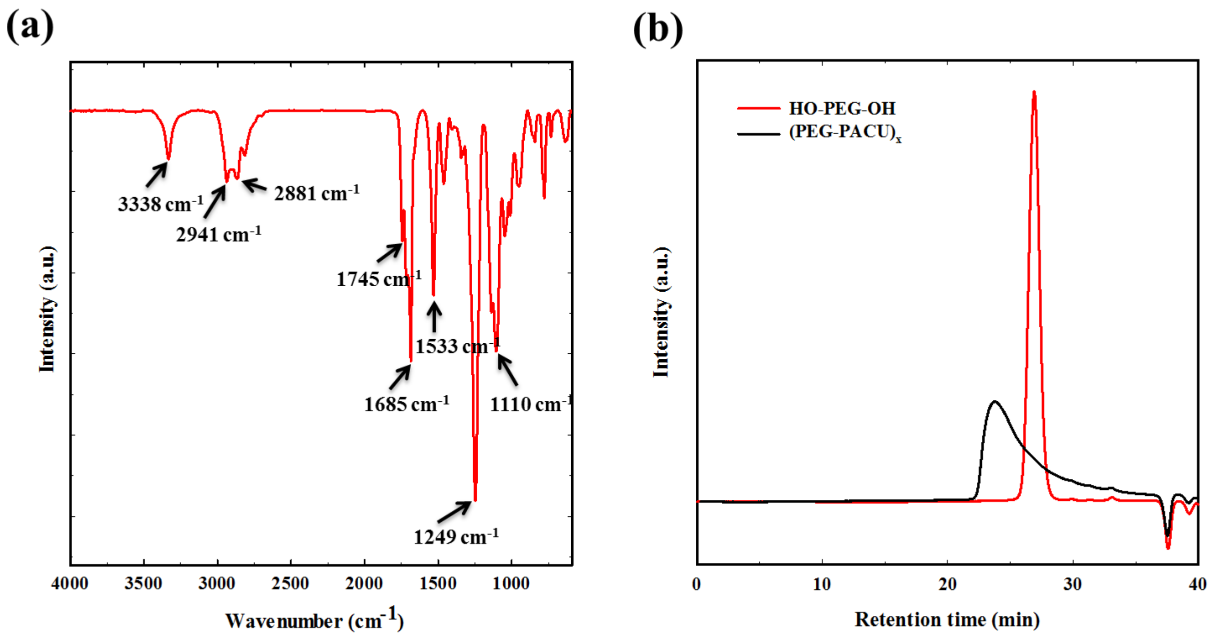

Fig. S2. (a) FT-IR spectra of PEG-PACU copolymers. (b) GPC trace of PEG-PACU copolymers. PEG was used as a standard.

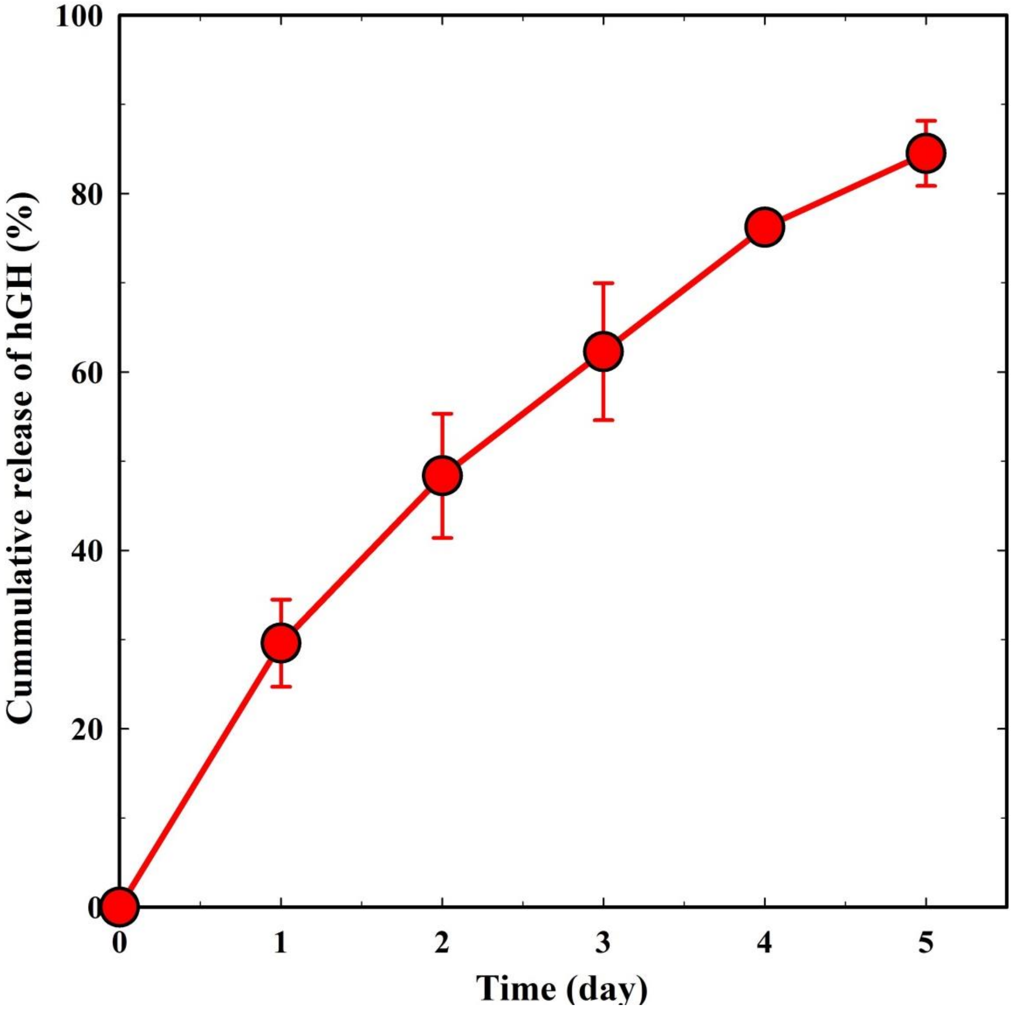

Fig. S3. In vitro release of hGH from PEG-PACU hydrogels (20 wt%) at a hGH concentration of 1 mg/ml. The error bars in the graph represent standard deviations (n=3).

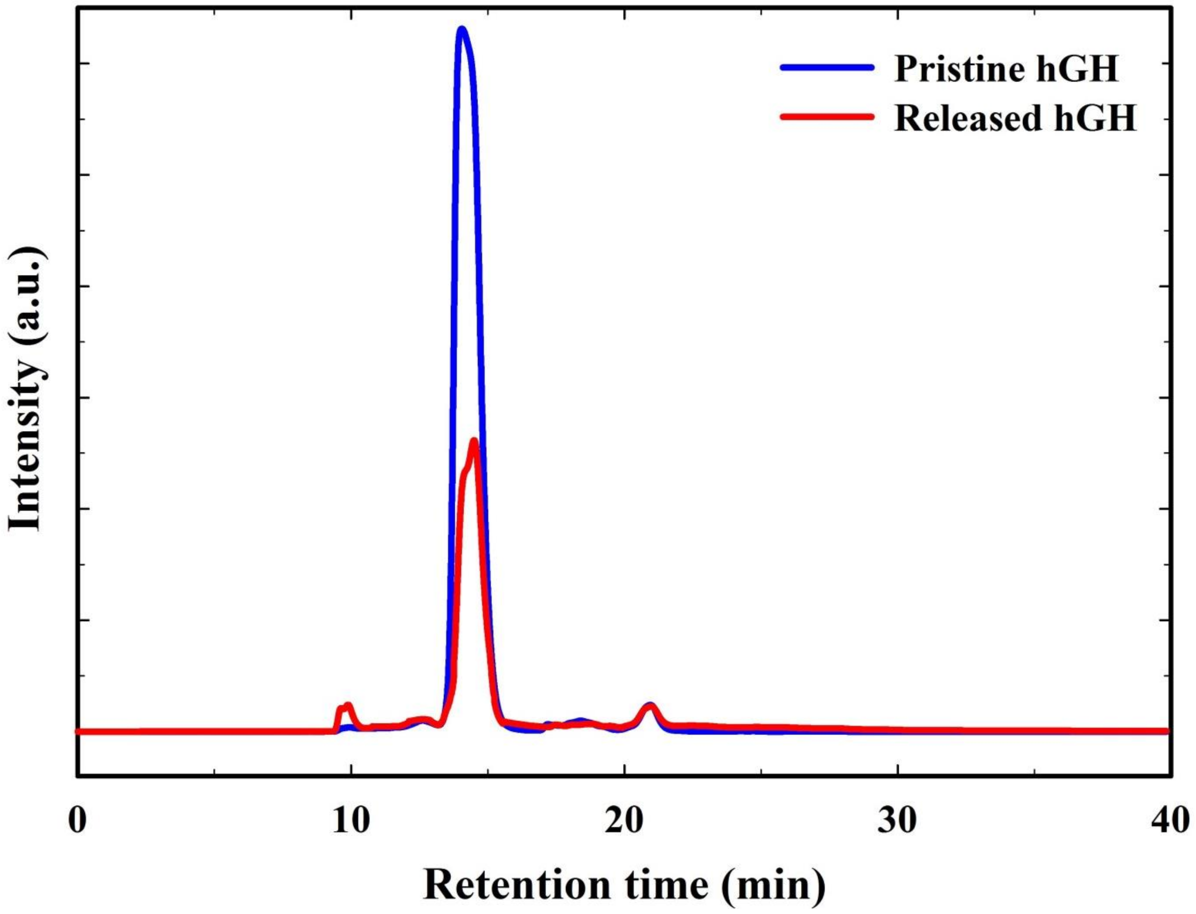

Fig. S4. HPLC chromatogram of pristine hGH and released hGH.

Table S1. Pharmacokinetic characteristics of hGH solution and hGH-loaded injectable hydrogels.

Formulation	T_{max} (h)	C_{max} (ng/mL)	AUC (ng.h/mL)
hGH solution	8	70.4	412
hGH-loaded PEG-PACU hydrogels	12	32.5	908
